# Free Recall Outperforms Story Recall in Associations with Plasma Biomarkers in Preclinical Alzheimer Disease

**DOI:** 10.14283/jpad.2024.130

**Published:** 2024-07-02

**Authors:** Andrew J. Aschenbrenner, J. J. Hassenstab, S. E. Schindler, S. Janelidze, O. Hansson, J. C. Morris, E. Grober

**Affiliations:** 1 Department of Neurology, Washington University, St. Louis, USA; 2 Clinical Memory Research Unit, Department of Clinical Sciences Malmö, Faculty of Medicine, Lund University, Lund, Sweden; 3 Memory Clinic, Skåne University Hospital, Malmö, Sweden; 4 Department of Neurology, Albert Einstein College of Medicine, New York, USA; 5 4488 Forest Park Ave, STE 301, St. Louis, MO 63108 USA

**Keywords:** Episodic memory, cognition, composite scores, plasma biomarkers, Alzheimer disease

## Abstract

**Background:**

A decline in episodic memory is one of the earliest cognitive characteristics of Alzheimer disease and memory tests are heavily featured in cognitive composite endpoints that are used to demonstrate treatment efficacy. Assessments of episodic memory can take many forms including free recall, associate learning, and paragraph or story recall. Plasma biomarkers of Alzheimer disease are now widely available and will likely form the backbone of cohort enrichment strategies for future clinical trials. Thus, it is critical to evaluate which episodic memory measures are most sensitive to plasma markers of Alzheimer disease pathology.

**Objectives:**

To compare the associations of common episodic memory tests with plasma biomarkers of Alzheimer disease.

**Design:**

Longitudinal cohort study.

**Setting:**

Academic medical center in the midwestern United States.

**Participants:**

A total of 161 cognitively normal older adults with at least one plasma biomarker assessment and two or more annual clinical and cognitive assessments which included up to three different tests of episodic memory.

**Measurements:**

Episodic memory performance using free recall, paired associates recall or paragraph recall. Plasma Aβ42, Aβ40, ptau217, and neurofilament light chain were measured.

**Results:**

Free recall on the Free and Cued Selective Reminding Test with Immediate Recall (FCSRT + IR) was substantially more sensitive to longitudinal cognitive change associated with abnormal baseline plasma Aβ42/Aβ40 and ptau217 compared to other measures of episodic memory. A cognitive composite that included only free recall showed larger decline associated with baseline Aβ42/Aβ40 when compared to those that included paragraph recall. Differences in decline across composites were minimal when considering baseline ptau217 or NfL.

**Conclusion:**

Episodic memory is a critical domain to assess in preclinical Alzheimer disease. Methods of assessing memory are not equal and longitudinal change in free recall substantially outperformed both paired associates and paragraph recall. Clinical trial results will depend critically on the episodic memory test(s) that are chosen for a composite endpoint and free recall from the FCSRT + IR is an optimal memory measure to include rather than paired associates or paragraph recall.

**Electronic Supplementary Material:**

Supplementary material is available in the online version of this article at 10.14283/jpad.2024.130.

## Introduction

Alzheimer disease (AD) is a progressive neurodegenerative disorder that is characterized by the abnormal accumulation of amyloid plaques and tau neurofibrillary tangles followed by neuronal loss. Both tau pathology and neuronal atrophy are most prominent in the medial temporal lobes and the hippocampus, brain regions involved in episodic memory (1–3). The primary clinical feature of AD is a progressive deterioration of cognitive ability, which often initially presents as an impairment in episodic memory (4). Cognitive tests used in the diagnosis of prodromal AD or mild cognitive impairment (MCI) due to AD emphasize tasks of episodic memory (5–6). In many cases, the degree of memory impairment strongly predicts who will progress to more severe stages of AD dementia (7–8).

AD pathology can accumulate for years before the onset of detectable clinical or cognitive symptoms (9). Biomarkers are now widely available to establish evidence of preclinical AD and include amyloid and tau PET imaging, cerebrospinal fluid markers of amyloid-β peptide (Aβ), tau, and tau phosphorylated at position 181 (p-tau181), and more recently, plasma biomarkers of similar analytes including tau phosphorylated at position 217 (p-tau217) (10–11). Individuals who are in the preclinical phase of the disease are at high risk of ultimately developing symptomatic AD (12–13) and therefore are increasingly targeted for enrollment in clinical trials. The rate of cognitive decline in preclinical AD is strongly associated with performance on episodic memory tasks (12, 14–17). As such, recently completed clinical trials in preclinical AD have emphasized episodic memory measures in their primary endpoints to demonstrate clinical efficacy of treatment (18–19).

Cognitive data can also be used cross-sectionally to enrich clinical trial cohorts for patients at high risk of progression. The original preclinical AD staging model (13) suggested that individuals with abnormal AD biomarkers coupled with subtle cognitive decline are at the highest risk of disease progression, a hypothesis that has been supported empirically (12), leading some trials, such as the A4 study (18), to require a certain score on an episodic memory test to be eligible for enrollment.

Despite the established importance of episodic memory in preclinical and prodromal AD, there is little consensus on how best to measure this construct. Indeed, memory measures vary widely in terms of their stimuli (e.g., single item words vs. paired associates vs. paragraphs vs. objects vs. faces), method of administration (e.g., number of learning trials, number of retrieval attempts, presence of semantic or phonological cues), method of testing (free recall, cued recall, recognition) and retention interval (immediate recall vs. delayed recall) to name just a few. Nevertheless, memory measures are often considered interchangeable. For example, the study validating the most popular general cognitive composite (ADCS-PACC) utilized different list learning measures in separate cohorts (20). This presumed equivalency is unwarranted as there are numerous demonstrations that one memory test (typically a list learning measure) is superior to another (typically paragraph recall) (15, 21).

The primary goal of this report is to compare three common memory tests in terms of their association with baseline differences in preclinical AD pathology. It is important to note that memory tests that may be highly sensitive to AD pathology at baseline may not be the same tests that are sensitive to longitudinal change, if, for example, there are confounding effects of practice-related improvements (22). Previous work examining the associations of cognitive composites with AD pathology has used CSF or neuroimaging biomarkers (23, 24). Recently, plasma biomarkers of AD pathology have become widely available and likely will form the basis of a cost-effective cohort enrichment strategy for future clinical trials that additionally make serial assessments more feasible (25–27). Therefore, in this report we focus on plasma biomarkers of amyloid pathology (the Aβ42/40 ratio), both amyloid and tau pathology (p-tau217), and neurodegeneration (neurofilament light chain [NfL]) (28–31).

A secondary goal was to compare the sensitivity of plasma biomarkers to decline in cognitive composite scores that employ the different memory tests available. Cognitive composites are now accepted as primary endpoints in secondary treatment trials (19, 32), and could be optimized by including only particularly sensitive memory tests, or conversely, by excluding poorly performing measures. If plasma biomarkers are to form the backbone of clinical trial recruitment, it is essential to establish the specific cognitive tests and composite scores that are most strongly associated with these measures of AD pathology.

## Methods

### Participants

This study analyzed longitudinal cognitive data from participants in an ongoing study of memory and aging at the Knight Alzheimer Disease Research Center in Washington University in St. Louis. Participants are typically recruited via referrals, outreach events hosted by the study team, and word of mouth. Study volunteers can range in age and may be cognitively healthy or have varying levels of cognitive impairment, but for the current analyses we restricted the sample as described below to best meet the goals of this study. All participants provided informed consent to participate in these studies and study procedures were conducted in accordance with the Declaration of Helsinki. To be included in the present analysis, participants must have been 65 years of age or older and clinically normal at baseline. Furthermore, they were required to have had measurements of all 3 plasma biomarkers (Aβ42/40 ratio, p-tau217, and NfL) within 2 years of their baseline cognitive visit, and to have at least 1 additional follow-up cognitive assessment. To avoid our statistical models being overly influenced by a few participants who have extremely long periods of follow-up, we restricted our follow-up data to a maximum of 10 years. Finally, due to COVID era closures, the cognitive battery was disrupted between 2020 and 2022. Some measures were temporarily dropped, and others were converted to an online administration format. Thus, we limit our analyses to data that was collected prior to the year 2020. Our final sample consisted of 161 cognitively healthy older adults and relevant demographic information on this cohort is presented in Table 1.

**Table 1:** Participant characteristics at the baseline cognitive visit

Variable	
N	161
Age (years)	72.0 (5.0)
Education (years)	16.5 (2.5)
Race	
Black / African American	14 (9%)
White	146 (91%)
Multiracial	1 (< 1%)
Sex	
Female	83 (52%)
Male	78 (48%)
APOE *ε*4 status	
Carrier	48 (30%)
Noncarrier	110 (68%)
Missing	3 (2%)
Mini-Mental State Exam	29.2 (1.1)
Interval between blood draw and baseline cognitive visit (days)	83.9 (36.1)
Number of visits	4.3 (2.0)
Cognitive Follow-up (years)	3.8 (2.1)
Plasma Aβ42/ Aβ40	0.10 (0.01)
Plasma p-tau 217	0.20 (0.12)
Plasma NfL (log)	2.5 (0.44)

Note: Continuous variables are summarized with the mean (SD) and categorical variables as N (%).

### Clinical and cognitive assessments

The presence of clinical dementia symptoms is established using the Clinical Dementia Rating® (CDR®) where a rating of 0 indicates the absence of symptoms (33). Participants in the current sample were all rated CDR 0 at their baseline assessment. A comprehensive cognitive battery is also administered annually which covers a wide range of cognitive domains including memory, attention, language, and processing speed (24). Our primary interest is on the memory tests for which we compare three common measures: free recall (FR) using the picture version of the Free and Cued Selective Reminding Test with Immediate Recall (FCSRT+IR) (34), paired associates (PA) recall from the Wechsler Memory Scale (35), and paragraph recall (PR) either from Wechsler Memory Scale Revised (36) or from the Craft Story recall test (37). For our second analytical goal, we developed two cognitive composites consisting of the Digit Symbol Substitution Test, Trail Making B, Category Fluency for Animals and either FR only (PACC-FR) or both FR and PR (PACC-FR-PR) included as measures of episodic memory. MMSE was not included in the PACCs since composites that include it are less sensitive in detecting Aβ-related cognitive decline in Aβ+ individuals (17).

### Plasma collection and processing

The plasma collection and processing protocol has been previously described (28). Briefly, blood was collected at 8 AM following an overnight fast. Aβ42 and Aβ40 were measured by C2N Diagnostics using an immunoprecipitation-mass spectrometry assay (38), p-tau217 was measured with the Lilly-developed assay at Lund University (31), and NfL was assessed with Quanterix Nf-Light assay kits at Washington University.

### Statistical Analysis

To enable comparison across the different memory measures, all cognitive tests were z-scored to the mean and standard deviation of the sample at baseline. For each cognitive outcome, linear mixed effects models were constructed using the lme4 package (39) in the R statistical computing environment, version 4.3.1, with baseline age, education, gender, years in study (hereafter referred to as “time”), a plasma biomarker, and the biomarker by time interaction included as fixed effects and random intercepts and slopes of time across participants. FR, PA, PR and the two cognitive composites were used as outcomes in separate models. Separate models were generated including either the plasma Aβ42/ Aβ40 ratio, p-tau217, or NfL. Outcomes are reported as a mean estimate with an associated 95% confidence interval. D-scores are provided as a measure of effect size and were calculated using the EMAtools (40) package in R. The d scores of longitudinal change (i.e., the biomarker by time interaction) are shown in Table 2 for each plasma biomarker and each cognitive outcome^1^.

**Table 2:** d-scores for longitudinal change in each outcome for each biomarker

	**Aβ42/ Aβ40**	**p-tau217**	**NfL**
pFCSRT+IR (FR)	0.59*	−0.51*	−0.37
Paragraph Recall (PR)	−0.25	−0.21	−0.19
Paired Associates (PA)	0.30	−0.42*	−0.40
PACC-FR	0.51*	−0.79*	−0.36*
PACC-FR-PR	0.30	−0.75*	−0.38*

Note: The d-scores were calculated as (2*t-value)/ sqrt(df) using the EMAtools package in R. Asterisks denote statistical significance at p < 0.05.

### Data availability policy

Data are available upon an approved request to the Knight ADRC (https://knightadrc.wustl.edu/Research/ResourceRequest.htm).

## Results

### Plasma Aβ42/40 results

**Figure 1:**
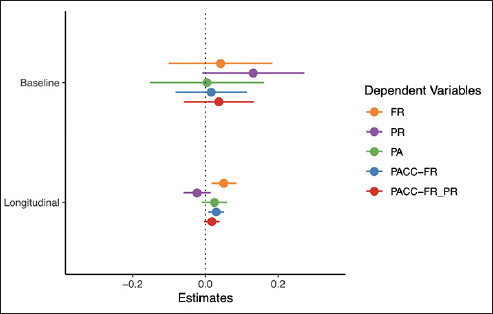
Results from the plasma Aβ42/ Aβ40 models Points are the regression estimate with a 95% confidence interval. Baseline = the cross-sectional difference associated with plasma Aβ42/ Aβ40, Longitudinal = the decline associated with baseline amyloid (i.e., the plasma Aβ42/ Aβ40 by time interaction). FR = Free recall from the Free and Cued Selective Reminding Test, PR = paragraph recall, PA = paired associates.

A summary of the plasma Aβ42/ 40 models is presented in Figure 1 (full model output and additional figures are provided in the Supplement). As shown, none of the cognitive tests or composite scores were associated with plasma Aβ42/40 at baseline (top panel). However, the rate of change in FR was associated with baseline plasma Aβ42/40 (the ratio by time interaction) with a relatively large effect size (beta = 0.05, CI = [0.02, 0.08], p = 0.004, d = 0.59). In contrast, the rate of change in PR and PA were not associated with baseline plasma Aβ42/Aβ40. The rate of change in a global cognitive composite that includes FR as the memory measure (PACC-FR) was more strongly associated with baseline plasma Aβ42/40 (beta = 0.03, CI = [0.01, 0.05], p = 0.007, d = 0.51) than a PACC score (PACC-FR-PR) that included both FR and PR (beta = 0.02, CI = [−0.00, 0.04], p = 0.105, d = 0.30). Thus, in this sample, the rate of change in FR alone was most strongly associated with baseline plasma Aβ42/Aβ40.

### Plasma p-tau217 results

**Figure 2:**
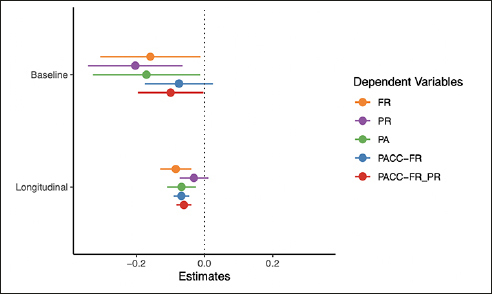
Results from the p-tau217 models Points are the regression estimate with a 95% confidence interval. Baseline = the cross-sectional difference associated with p-tau217, Longitudinal = the decline associated with p-tau217 (i.e., the p-tau217 by time interaction). FR = Free recall from the Free and Cued Selective Reminding Test, PR = paragraph recall, PA = paired associates.

The plasma p-tau217 models are summarized in Figure 2 (full model output and additional figures are available in the Supplement). As shown in the top panel, all cognitive tests were associated with p-tau217 at baseline with the exception of PACC-FR composite with small to moderate effect sizes (ds, FR= −0.32, PR = −0.45, PA = −0.33, PACC-FR-PR = −0.32). Similarly, the rates of change in FR and PA (but not PR), were associated with baseline p-tau217 levels. This decline was largest for FR (beta = −0.08, CI = [−0.13, −0.04], d = −0.51) followed by PA (beta = −0.07, CI = [−0.11, −0.02], d = −0.42). Rates of change for cognitive composite scores were relatively similarly associated with baseline p-tau217 (PACC-FR: beta = −0.07, CI = [−0.09, −0.05], d = −0.79; PACC-FR-PR: beta = −0.06, CI = [−0.08, −0.04], d = −0.76).

### NfL results

**Figure 3:**
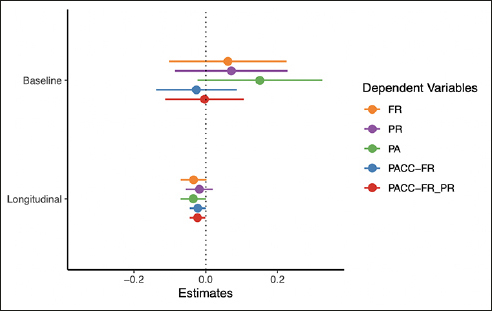
Results from the NfL models Points are the regression estimate with a 95% confidence interval. Baseline = the cross-sectional difference associated with NfL, Longitudinal = the decline associated with NfL (i.e., the NfL by time interaction). FR = Free recall from the Free and Cued Selective Reminding Test, PR = paragraph recall, PA = paired associates.

A summary of the plasma NfL models is presented in Figure 3 (full model output is available in the supplement materials). As with the plasma Aβ42/40 models, none of the memory tests nor the cognitive composites were associated with NfL at baseline. Rates of change for FR and PA had similar associations with baseline plasma NfL (FR: beta = −0.03, CI = [−0.07, 0.00], d = −0.37; PA: beta = −0.03, CI = [−0.07, 0.00], d = −0.40). Furthermore, rates of change for both cognitive composites were similarly associated with baseline plasma NfL (PACC-FR: beta = −0.02, CI = [−0.04, −0.00], d = −0.36; PACC-FR-PR: beta = PACC-FR: beta = −0.02, CI = [−0.04, −0.00], d = −0.38).

## Discussion

Due to their cost effectiveness, ease of collection, and concordance with other markers of AD pathology, plasma biomarkers are poised to become the primary cohort enrichment strategy for clinical trials on AD. Cross-sectional impairment and longitudinal decline in episodic memory is consistently one of most sensitive cognitive signals of AD. It is critical, therefore, to establish which specific memory measures are the most strongly correlated with plasma biomarkers. We discuss our findings centered around several key points.

First, at baseline, there was no association between plasma Aβ42/40 or NfL and any memory test. However, all three memory tests were associated with p-tau217, with the strongest effect appearing on paragraph recall. Plasma p-tau217 reflects both amyloid and tau pathology (30, 42, 43), and tau pathology is more strongly associated with cognitive impairment than amyloid pathology alone (44,45). Second, there were clear dissociations among the memory measures when considering longitudinal change. There was a strong association between decline in the pFCSRT+IR and plasma Aβ42/40 (d=0.59), which was not seen for paragraph recall and paired associates recall (ds = −0.25 and 0.30, respectively). Other studies have also reported that FR outperformed paragraph recall in predicting incident AD or biomarker profiles (21, 46, 47). Although the differences were more modest, decline in pFCSRT+IR was more strongly associated with baseline p-tau217 as compared to PA; the decline in FR and PA had similar associations with baseline plasma NfL. The poor longitudinal performance of the paragraph recall tests is possibly due the pronounced practice effect relative to list learning tests (22, 48). The additional advantage of the pFCSRT+IR over PA is likely due in part to the fact that pFCSRT+IR controls attention and semantic encoding during acquisition to maximize recall and PA does not.

Although preclinical AD can be diagnosed in the absence of cognitive impairment, evidence of cognitive decline has become an important outcome for investigating the prognostic efficacy of plasma biomarkers. We focused on episodic memory because impairment on episodic memory tasks is the hallmark cognitive deficit of AD and occurs early in the disease course. When preclinical AD was first described, cognitive impairment was thought to occur after β-amyloid plaque deposition and neurofibrillary tau aggregation pathology (13). An important question is when in preclinical AD does amyloid accumulation become associated with cognitive impairment (49).

Evidence of FR sensitivity to early biomarker changes in preclinical AD can be gleaned from clinical and biomarker studies. The early emergence of FR impairment as a predictor of symptomatic AD has been observed in longitudinal cohort studies in the US and Europe. Recently, we have identified a subset of cognitively normal participants who have impaired FR in several cohort studies including the Knight ADRC (18.1%), HABS (15%), A4 (20%) and the BLSA (16%) (50–53). Using the assessment closest to death, 300+ cases from the clinicopathological series from the Knight ADRC were classified into Braak stages (54). FR scores were lower in cases at Braak stage III compared to Braak stages 0 and I (combined) while MMSE and CDR scores for individuals did not differ from Braak stages 0/I until Braak stage IV.

Of course, it is now standard practice to examine decline on cognitive composite scores as opposed to single tests. Nevertheless, the specific tests selected to comprise the final composite will have a critical bearing on the final results. For example, the pFCSRT+IR is one component of the PACC in the HABS cohort that also included paragraph recall, digit symbol substitution, and the MMSE. When FR was included in the PACC, differences between +/− Aβ groups emerged earlier then when FR was not included over 3 and 5 years of follow-up (55). In the A4 cohort, the magnitude of the decrease in FR at subclinical levels of Aβ compared to normal levels was more than twice that of the other PACC components and with a larger effect size than the PACC (49). These results mirror the present study, where a cognitive composite that included only FR substantially outperformed the composite that included both FR and PR. This benefit of an FR only composite was specifically associated with baseline plasma Aβ42/40, as both composite scores were similarly sensitive to cognitive decline that was associated with baseline plasma p-tau and NfL.

It was unexpected that rate of change in FR was so strongly associated with baseline plasma Aβ42/40. This may indicate the temporal relationship between plasma Aβ42/40 and FR: that Aβ42/40 changes shortly before FR starts to decline. Global and theoretically derived cognitive composites exhibited stronger associations with the interaction of age and plasma Aβ42/40 levels than empirically derived memory composites or raw scores from single memory tests including story recall, a list learning test, and a visual memory test (23). Both list learning (AVLT) and story recall (LM) exhibited insignificant biomarker associations individually but when combined with a visual memory test that itself was significantly associated with the biomarker, the association of the composite was enhanced unlike what we observed in the current study. Nevertheless, episodic memory declines assessed by FR occur earliest in preclinical AD with executive functioning declining several years later (16). It is possible that non-memory aspects of standard cognitive composites are more strongly related to tau pathology, which occurs later and is not indexed by plasma Aβ42/40 as shown by the increased sensitivity to tau when executive function tests are combined with FR. A recent study found that plasma Ab42/40 changed 5 years earlier than a measure of plasma p-tau217 (56). When tau pathology begins to also accumulate, as reflected by p-tau217, the predictive utility of a cognitive composite becomes greatly enhanced. We see this as another demonstration that the selection of specific tests to be used in composites can be critical to the measurement of its associations with biomarkers and clinical progression.

The PACC-FR-PR in the current study is similar to the Z-scores of Attention, Verbal fluency, and Episodic memory for Nondemented older adults (ZAVEN) composite because the composite tests focus on memory and executive function (17). The ZAVEN is comprised of the DSST, FAS, story recall, and the CVLT instead of the pFCSRT+IR. Over 6 years of follow-up, cognitively normal participants in the AIBL cohort with high (SUVR >1.90 and intermediate (1.50–1.90) levels of amyloid burden showed greater cognitive progression when measured by the ZAVEN than other composites that either included the MMSE or did not include measures of executive function. Even amyloid burden levels under CL40, a composite measure of executive functioning/processing speed and memory retrieval tasks provided the strongest prediction of decline in the HABS cohort, while PACC score remained optimal at high levels of Aβ (>CL40) (57).

Despite the many strengths of this study including a large, well-characterized cohort and many years of repeated testing, there are some limitations that should be noted. First, our sample is highly educated, and the majority of the sample self-identified as White. This may limit generalizability of the findings to the larger population, especially if there are differences across race in biomarker levels (58,59), or cognitive test scores (60,61) due to differences in social or environmental factors. It will be important in future analyses to consider social determinants of health and other factors that may modify the relationships shown in the present work. Additionally, we examined only linear rates of change, and it may be fruitful to also consider non-linear trajectories in subsequent analyses.

## Conclusion

Plasma Aβ42/40, p-tau217, and NfL are strong predictors of FR decline in preclinical AD. Caution is recommended when combining components in cognitive composites, particularly when considering decline that may be associated with plasma Aβ42/40. Combining FR with story recall may weaken the association with plasma Aβ42/40, thereby reducing their prognostic value. Episodic memory and executive function are important domains to be assessed in preclinical AD; which tests are used in their measurement may affect the magnitude of associations with biomarkers and clinical progression. Our results suggest that FR may be an ideal test to consider when monitoring longitudinal changes in memory. While FR decline on the pFCSRT+IR may mark the start of episodic memory impairment in preclinical AD, other methods like those that include daily brief repeated memory testing may reveal impairment at an even earlier point (62, 63).

## Electronic supplementary material

Supplementary material, approximately 138 KB.

## Footnotes

1 1. It should be noted that the calculation of effect size metrics in linear mixed effects models is not straightforward and is subject to some controversy41. The formula for effect size used in this report is noted in Table {xiTab2|2}. It isn’t clear how well this formulation will compare to traditional calculations of Cohen’s d and is best considered as a way to combine magnitude of the effect with its variability and error. This will assist comparison of effects across the tasks used in this analysis but may not compare directly to other analyses from other statistical models. Raw regression weights are provided in the text and supplementary materials which are also useful for comparison.

## Notes

*Ethical standards:* All participants provided written informed consent to participate in these studies and all procedures were approved by the Institutional Review Board at Washington University in St. Louis.

## References

[1] Jack CR, Petersen RC, O’Brien PC, Tangalos EG. MR-based hippocampal volumetry in the diagnosis of Alzheimer’s disease. Neurology. 1992;42(1):183–183. doi:10.1212/WNL.42.1.183 1734300 10.1212/wnl.42.1.183

[2] Price JL, Ko AI, Wade MJ, Tsou SK, McKeel DW, Morris JC. Neuron number in the entorhinal cortex and CA1 in preclinical Alzheimer disease. Arch Neurol. 2001;58(9):1395. doi:10.1001/archneur.58.9.1395 11559310 10.1001/archneur.58.9.1395

[3] Squire LR. Memory and the hippocampus: A synthesis from findings with rats, monkeys, and humans. Psychological Review. 1992;99(2):195–231. doi:10.1037/0033-295X.99.2.195 1594723 10.1037/0033-295x.99.2.195

[4] Albert MS. Changes in cognition. Neurobiology of Aging. 2011;32:S58–S63. doi:10.1016/j.neurobiolaging.2011.09.010 22078174 10.1016/j.neurobiolaging.2011.09.010 PMC3929949

[5] Albert MS, DeKosky ST, Dickson D, et al. The diagnosis of mild cognitive impairment due to Alzheimer’s disease: Recommendations from the National Institute on Aging-Alzheimer’s Association workgroups on diagnostic guidelines for Alzheimer’s disease. Alzheimer’s & Dementia. 2011;7(3):270–279. doi:10.1016/j.jalz.2011.03.008 10.1016/j.jalz.2011.03.008 PMC3312027 21514249

[6] Petersen RC. MCI Criteria in ADNI: Meeting Biological Expectations. Neurology. 2021;97(12):597–599. doi:10.1212/WNL.0000000000012588 34341151 10.1212/WNL.0000000000012588 PMC8480480

[7] Dierckx E, Engelborghs S, De Raedt R, et al. Verbal cued recall as a predictor of conversion to Alzheimer’s disease in Mild Cognitive Impairment. Int J Geriat Psychiatry. 2009;24(10):1094–1100. doi:10.1002/gps.2228 10.1002/gps.2228 19280679

[8] Dubois B, Albert ML. Amnestic MCI or prodromal Alzheimer’s disease? The Lancet Neurology. 2004;3(4):246–248. doi:10.1016/S1474-4422(04)00710-0 15039037 10.1016/S1474-4422(04)00710-0

[9] Bateman RJ, Xiong C, Benzinger TL, et al. Clinical and biomarker changes in Dominantly Inherited Alzheimer’s disease. New England Journal of Medicine. 2012;367(9):795–804. doi:10.1056/NEJMoa1202753 22784036 10.1056/NEJMoa1202753 PMC3474597

[10] Hansson O. Biomarkers for neurodegenerative diseases. Nat Med. 2021;27(6):954–963. doi:10.1038/s41591-021-01382-x 34083813 10.1038/s41591-021-01382-x

[11] Hansson O, Blennow K, Zetterberg H, Dage J. Blood biomarkers for Alzheimer’s disease in clinical practice and trials. Nat Aging. 2023;3(5):506–519. doi:10.1038/s43587-023-00403-3 37202517 10.1038/s43587-023-00403-3 PMC10979350

[12] Vos SJ, Xiong C, Visser PJ, et al. Preclinical Alzheimer’s disease and its outcome: a longitudinal cohort study. The Lancet Neurology. 2013;12(10):957–965. doi:10.1016/S1474-4422(13)70194-7 24012374 10.1016/S1474-4422(13)70194-7 PMC3904678

[13] Sperling RA, Aisen PS, Beckett LA, et al. Toward defining the preclinical stages of Alzheimer’s disease: Recommendations from the National Institute on Aging-Alzheimer’s Association workgroups on diagnostic guidelines for Alzheimer’s disease. Alzheimer’s & Dementia. 2011;7(3):280–292. doi:10.1016/j.jalz.2011.03.003 10.1016/j.jalz.2011.03.003 PMC3220946 21514248

[14] Lim YY, Pietrzak RH, Ellis KA, et al. Rapid decline in episodic memory in healthy older adults with high amyloid-β. JAD. 2013;33(3):675–679. doi:10.3233/JAD-2012-121516 23001710 10.3233/JAD-2012-121516

[15] Johnson DK, Storandt M, Morris JC, Galvin JE. Longitudinal study of the transition from healthy aging to Alzheimer disease. Archives of Neurology. 2009;66(10). doi:10.1001/archneurol.2009.158 10.1001/archneurol.2009.158 PMC2795328 19822781

[16] Grober E, Hall CB, Lipton RB, Zonderman AB, Resnick SM, Kawas C. Memory impairment, executive dysfunction, and intellectual decline in preclinical Alzheimer’s disease. J Inter Neuropsych Soc. 2008;14(02). doi:10.1017/S1355617708080302 10.1017/S1355617708080302 PMC2763488 18282324

[17] Lim YY, Snyder PJ, Pietrzak RH, et al. Sensitivity of composite scores to amyloid burden in preclinical Alzheimer’s disease: Introducing the Z-scores of Attention, Verbal fluency, and Episodic memory for Nondemented older adults composite score. Alz & Dem Diag Ass & Dis Mo. 2016;2(1):19–26. doi:10.1016/j.dadm.2015.11.003 10.1016/j.dadm.2015.11.003 PMC4879646 27239532

[18] Sperling RA, Rentz DM, Johnson KA, et al. The A4 study: Stopping AD before symptoms begin? Science Translational Medicine. 2014;6(228):228fs13–228fs13. doi:10.1126/scitranslmed.3007941 24648338 10.1126/scitranslmed.3007941 PMC4049292

[19] Bateman RJ, Benzinger TL, Berry S, et al. The DIAN-TU Next Generation Alzheimer’s prevention trial: Adaptive design and disease progression model. Alzheimer’s & Dementia. 2017;13(1):8–19. doi:10.1016/j.jalz.2016.07.005 10.1016/j.jalz.2016.07.005 PMC5218895 27583651

[20] Donohue MC, Sperling RA, Salmon DP, et al. The Preclinical Alzheimer Cognitive Composite: Measuring Amyloid-Related Decline. JAMA Neurology. 2014;71(8):961. doi:10.1001/jamaneurol.2014.803 24886908 10.1001/jamaneurol.2014.803 PMC4439182

[21] Grober E, Mowrey W, Katz M, Derby C, Lipton RB. Conventional and robust norming in identifying preclinical dementia. Journal of Clinical and Experimental Neuropsychology. 2015;37(10):1098–1106. doi:10.1080/13803395.2015.1078779 26325449 10.1080/13803395.2015.1078779 PMC6790124

[22] Aschenbrenner AJ, Hassenstab J, Wang G, et al. Avoid or embrace? Practice effects in alzheimer’s disease prevention trials. Front Aging Neurosci. 2022;14:883131. doi:10.3389/fnagi.2022.883131 35783127 10.3389/fnagi.2022.883131 PMC9244171

[23] Jonaitis EM, Koscik RL, Clark LR, et al. Measuring longitudinal cognition: Individual tests versus composites. Alz & Dem Diag Ass & Dis Mo. 2019;11(1):74–84. doi:10.1016/j.dadm.2018.11.006 10.1016/j.dadm.2018.11.006 PMC6816509 31673596

[24] McKay NS, Millar PR, Nicosia J, et al. Pick a PACC: Comparing domain-specific and general cognitive composites in Alzheimer disease research. Neuropsychology. Published online April 11, 2024. doi:10.1037/neu0000949 10.1037/neu0000949 PMC11176005 38602816

[25] Mattsson-Carlgren N, Collij LE, Stomrud E, et al. Plasma biomarker strategy for selecting patients with alzheimer disease for antiamyloid immunotherapies. JAMA Neurol. Published online December 4, 2023. doi:10.1001/jamaneurol.2023.4596 10.1001/jamaneurol.2023.4596 PMC10696515 38048096

[26] Schindler SE, Li Y, Li M, et al. Using Alzheimer’s disease blood tests to accelerate clinical trial enrollment. Alzheimer’s & Dementia. 2023;19(4):1175–1183. doi:10.1002/alz.12754 10.1002/alz.12754 PMC9902574 35934777

[27] Ashton NJ, Janelidze S, Mattsson-Carlgren N, et al. Differential roles of Aβ42/40, p-tau231 and p-tau217 for Alzheimer’s trial selection and disease monitoring. Nat Med. 2022;28(12):2555–2562. doi:10.1038/s41591-022-02074-w 36456833 10.1038/s41591-022-02074-w PMC9800279

[28] Schindler SE, Bollinger JG, Ovod V, et al. High-precision plasma β-amyloid 42/40 predicts current and future brain amyloidosis. Neurology. 2019;93(17):e1647–e1659. doi:10.1212/WNL.0000000000008081 31371569 10.1212/WNL.0000000000008081 PMC6946467

[29] West T, Kirmess KM, Meyer MR, et al. A blood-based diagnostic test incorporating plasma Aβ42/40 ratio, ApoE proteotype, and age accurately identifies brain amyloid status: findings from a multi cohort validity analysis. Mol Neurodegeneration. 2021;16(1):30. doi:10.1186/s13024-021-00451-6 10.1186/s13024-021-00451-6 PMC8088704 33933117

[30] Ashton NJ, Brum WS, Di Molfetta G, et al. Diagnostic accuracy of a plasma phosphorylated tau 217 immunoassay for Alzheimer disease pathology. JAMA Neurol. Published online January 22, 2024. doi:10.1001/jamaneurol.2023.5319 10.1001/jamaneurol.2023.5319 PMC10804282 38252443

[31] Palmqvist S, Janelidze S, Quiroz YT, et al. Discriminative accuracy of plasma phospho-tau217 for Alzheimer disease vs other neurodegenerative disorders. JAMA. 2020;324(8):772. doi:10.1001/jama.2020.12134 32722745 10.1001/jama.2020.12134 PMC7388060

[32] Rafii MS, Sperling RA, Donohue MC, et al. The AHEAD 3–45 Study: Design of a prevention trial for Alzheimer’s disease. Alzheimer’s & Dementia. 2023;19(4):1227–1233. doi:10.1002/alz.12748 10.1002/alz.12748 PMC9929028 35971310

[33] Morris JC. The Clinical Dementia Rating (CDR): Current version and scoring rules. Neurology. 1993;43(11):2412.2–2412–a. doi:10.1212/WNL.43.11.2412-a 10.1212/wnl.43.11.2412-a 8232972

[34] Grober E, Lipton RB, Hall C, Crystal H. Memory impairment on free and cued selective reminding predicts dementia. Neurology. 2000;54(4):827–832. doi:10.1212/WNL.54.4.827 10690971 10.1212/wnl.54.4.827

[35] Wechsler D, Stone C. Manual: Wechsler Memory Scale. Psychological Corporation; 1973.

[36] Wechsler D. Manual: Wechsler Memory Scale-Revised. Psychological Corporation; 1987.

[37] Craft S, Newcomer J, Kanne S, et al. Memory improvement following induced hyperinsulinemia in alzheimer’s disease. Neurobiology of Aging. 1996;17(1):123–130. doi:10.1016/0197-4580(95)02002-0 8786794 10.1016/0197-4580(95)02002-0

[38] Kirmess KM, Meyer MR, Holubasch MS, et al. The PrecivityADTM test: Accurate and reliable LC-MS/MS assays for quantifying plasma amyloid beta 40 and 42 and apolipoprotein E proteotype for the assessment of brain amyloidosis. Clinica Chimica Acta. 2021;519:267–275. doi:10.1016/j.cca.2021.05.011 10.1016/j.cca.2021.05.011 PMC10292789 34015303

[39] Bates D, Mächler M, Bolker B, Walker S. Fitting linear mixed-effects models using lme4. Journal of Statistical Software. 2015;67(1):1–48. doi:10.18637/jss.v067.i01

[40] Kleiman E. EMAtools: Data management tools for real-time monitoring/ecological momentary assessment data. Published online 2021. https://CRAN.R-project.org/package=EMAtools

[41] Westfall J, Kenny DA, Judd CM. Statistical power and optimal design in experiments in which samples of participants respond to samples of stimuli. Journal of Experimental Psychology: General. 2014;143(5):2020–2045. doi:10.1037/xge0000014 25111580 10.1037/xge0000014

[42] Barthélemy NR, Salvadó G, Schindler SE, et al. Highly accurate blood test for Alzheimer’s disease is similar or superior to clinical cerebrospinal fluid tests. Nat Med. Published online February 21, 2024. doi:10.1038/s41591-024-02869-z 10.1038/s41591-024-02869-z PMC11031399 38382645

[43] Salvadó G, Ossenkoppele R, Ashton NJ, et al. Specific associations between plasma biomarkers and postmortem amyloid plaque and tau tangle loads. EMBO Mol Med. 2023;15(5):e17123. doi:10.15252/emmm.202217123 36912178 10.15252/emmm.202217123 PMC10165361

[44] Barthélemy NR, Saef B, Li Y, et al. CSF tau phosphorylation occupancies at T217 and T205 represent improved biomarkers of amyloid and tau pathology in Alzheimer’s disease. Nat Aging. 2023;3(4):391–401. doi:10.1038/s43587-023-00380-7 37117788 10.1038/s43587-023-00380-7 PMC10154225

[45] Ossenkoppele R, Pichet Binette A, Groot C, et al. Amyloid and tau PET-positive cognitively unimpaired individuals are at high risk for future cognitive decline. Nat Med. 2022;28(11):2381–2387. doi:10.1038/s41591-022-02049-x 36357681 10.1038/s41591-022-02049-x PMC9671808

[46] Derby CA, Burns LC, Wang C, et al. Screening for predementia AD: Time-dependent operating characteristics of episodic memory tests. Neurology. 2013;80(14):1307–1314. doi:10.1212/WNL.0b013e31828ab2c9 23468542 10.1212/WNL.0b013e31828ab2c9 PMC3656458

[47] Wagner M, Wolf S, Reischies FM, et al. Biomarker validation of a cued recall memory deficit in prodromal Alzheimer disease. Neurology. 2012;78(6):379–386. doi:10.1212/WNL.0b013e318245f447 22238414 10.1212/WNL.0b013e318245f447

[48] Gavett BE, Gurnani AS, Saurman JL, et al. Practice effects on story memory and list learning tests in the neuropsychological assessment of older adults. Ito E, ed. PLoS ONE. 2016;11(10):e0164492. doi:10.1371/journal.pone.0164492 27711147 10.1371/journal.pone.0164492 PMC5053775

[49] Insel PS, Mormino EC, Aisen PS, Thompson WK, Donohue MC. Neuroanatomical spread of amyloid β and tau in Alzheimer’s disease: implications for primary prevention. Brain Communications. 2020;2(1):fcaa007. doi:10.1093/braincomms/fcaa007 32140682 10.1093/braincomms/fcaa007 PMC7048875

[50] Grober E, Petersen KK, Lipton RB, et al. Association of stages of objective memory impairment with incident symptomatic cognitive impairment in cognitively normal individuals. Neurology. 2023;100(22):e2279–e2289. doi:10.1212/WNL.0000000000207276 37076305 10.1212/WNL.0000000000207276 PMC10259282

[51] Grober E, Papp KV, Rentz DM, et al. Neuroimaging correlates of Stages of Objective Memory Impairment (SOMI) system. Alz & Dem Diag Ass & Dis Mo. 2021;13(1):e12224. doi:10.1002/dad2.12224 10.1002/dad2.12224 PMC8719429 35005192

[52] Grober E, Lipton RB, Sperling RA, et al. Associations of stages of objective memory impairment with amyloid pet and structural mri: the a4 study. Neurology. 2022;98(13):e1327–e1336. doi:10.1212/WNL.0000000000200046 35197359 10.1212/WNL.0000000000200046 PMC8967421

[53] Grober E, Wang C, Kitner-Triolo M, Lipton RB, Kawas C, Resnick SM. Prognostic value of learning and retention measures from the free and cued selective reminding test to identify incident mild cognitive impairment. J Int Neuropsychol Soc. 2022;28(3):292–299. doi:10.1017/S1355617721000291 33745492 10.1017/S1355617721000291 PMC8455713

[54] Grober E, Qi Q, Kuo L, Hassenstab J, Perrin RJ, Lipton RB. The free and cued selective reminding test predicts braak stage. JAD. 2021;80(1):175–183. doi:10.3233/JAD-200980 33492287 10.3233/JAD-200980 PMC8075386

[55] Mormino EC, Papp KV, Rentz DM, et al. Early and late change on the preclinical Alzheimer’s cognitive composite in clinically normal older individuals with elevated amyloid β. Alzheimer’s & Dementia. 2017;13(9):1004–1012. doi:10.1016/j.jalz.2017.01.018 10.1016/j.jalz.2017.01.018 PMC5573651 28253478

[56] Li Y, Yen D, Hendrix RD, et al. Timing of Biomarker Changes in Sporadic Alzheimer’s Disease in Estimated Years from Symptom Onset. Annals of Neurology. Published online February 24, 2024:ana.26891. doi:10.1002/ana.26891 10.1002/ana.26891 PMC11060905 38400792

[57] Farrell ME, Papp KV, Buckley RF, et al. Association of emerging β-amyloid and tau pathology with early cognitive changes in clinically normal older adults. Neurology. 2022;98(15). doi:10.1212/WNL.0000000000200137 10.1212/WNL.0000000000200137 PMC9012271 35338074

[58] Hajjar I, Yang Z, Okafor M, et al. Association of plasma and cerebrospinal fluid Alzheimer disease biomarkers with race and the role of genetic ancestry, vascular comorbidities, and neighborhood factors. JAMA Netw Open. 2022;5(10):e2235068. doi:10.1001/jamanetworkopen.2022.35068 36201209 10.1001/jamanetworkopen.2022.35068 PMC9539715

[59] Xiong C, Schindler S, Luo J, et al. Baseline levels and longitudinal rates of change in plasma Aβ42/40among self-identified Black/African American and White individuals. Published online January 8, 2024. doi:10.21203/rs.3.rs-3783571/v1

[60] Manly JJ, Jacobs DM, Touradji P, Small SA, Stern Y. Reading level attenuates differences in neuropsychological test performance between African American and White elders. J Int Neuropsychol Soc. 2002;8(3):341–348. doi:10.1017/S1355617702813157 11939693 10.1017/s1355617702813157

[61] Barnes LL, Yumoto F, Capuano A, Wilson RS, Bennett DA, Tractenberg RE. Examination of the factor structure of a global cognitive function battery across race and time. J Int Neuropsychol Soc. 2016;22(1):66–75. doi:10.1017/S1355617715001113 26563713 10.1017/S1355617715001113 PMC4763720

[62] Papp KV, Jutten RJ, Soberanes D, et al. Early detection of amyloid-related changes in memory among cognitively unimpaired older adults with daily digital testing. Annals of Neurology. Published online December 19, 2023:ana.26833. doi:10.1002/ana.26833 10.1002/ana.26833 PMC10922126 37991080

[63] Nicosia J, Aschenbrenner AJ, Balota DA, et al. Unsupervised high-frequency smartphone-based cognitive assessments are reliable, valid, and feasible in older adults at risk for Alzheimer’s disease. J Int Neuropsychol Soc. Published online September 5, 2022:1–13. doi:10.1017/S135561772200042X 10.1017/S135561772200042X PMC9985662 36062528

