# Supplementary material for: Free Recall Outperforms Story Recall in Associations with Plasma Biomarkers in Preclinical Alzheimer Disease
Source: J Prev Alzheimers Dis. 2024 Jul 2;11(6):1696–702. doi: 10.14283/jpad.2024.130 (PMC11573877; doi:10.14283/jpad.2024.130)
Supplement: Supplementary file 1 — Supplementary material, approximately 138 KB. [file 42414_2024_130_MOESM1_ESM.docx]

**Supplementary Tables and Figures**

Full model output for each cognitive outcome (Free Recall, Paragraph Recall, Paired Associates) by each plasma biomarker (Aβ42/ Aβ40, p-tau217 and NfL). The associated figure plots the longitudinal trajectory of each outcome at the mean biomarker and 1 SD above and below the mean.

|  | **Free Recall** | | | |
| --- | --- | --- | --- | --- |
| *Predictors* | *Estimates* | *CI* | *p* | *df* |
| (Intercept) | 1.58 | -0.83 – 4.00 | 0.198 | 668.00 |
| Aβ42/ Aβ40 | 0.04 | -0.10 – 0.18 | 0.568 | 668.00 |
| years | -0.01 | -0.05 – 0.02 | 0.504 | 668.00 |
| bl age | -0.02 | -0.05 – 0.00 | 0.097 | 668.00 |
| EDUC | -0.02 | -0.08 – 0.03 | 0.411 | 668.00 |
| GENDER | 0.38 | 0.10 – 0.66 | **0.008** | 668.00 |
| Aβ42/ Aβ40 × years | 0.05 | 0.02 – 0.08 | **0.004** | 668.00 |
| **Random Effects** | | | | |
| σ^2^ | 0.27 | | | |
| τ_00_ _ID_ | 0.65 | | | |
| τ_11_ _ID.years_ | 0.02 | | | |
| ρ_01_ _ID_ | 0.14 | | | |
| ICC | 0.77 | | | |
| N _ID_ | 160 | | | |
| Observations | 679 | | | |
| Marginal R^2^ / Conditional R^2^ | 0.086 / 0.788 | | | |

Figure 1. Rate of change in FCSRT free recall as a function of AB42/40 levels. Both the biomarker and the cognition scores were z-scored to baseline. Lines represent the mean value of the biomarker (blue line), 1 standard deviation below the mean (red line) and 1 standard deviation above the mean (green line).

|  | **Paragraph Recall** | | | |
| --- | --- | --- | --- | --- |
| *Predictors* | *Estimates* | *CI* | *p* | *df* |
| (Intercept) | 1.31 | -0.92 – 3.55 | 0.249 | 674.00 |
| Aβ42/ Aβ40 | 0.13 | -0.01 – 0.27 | 0.066 | 674.00 |
| years | 0.03 | -0.01 – 0.07 | 0.155 | 674.00 |
| bl age | -0.03 | -0.06 – -0.01 | **0.016** | 674.00 |
| EDUC | 0.03 | -0.02 – 0.08 | 0.201 | 674.00 |
| GENDER | 0.34 | 0.08 – 0.60 | **0.010** | 674.00 |
| Aβ42/ Aβ40 × years | -0.02 | -0.06 – 0.01 | 0.220 | 674.00 |
| **Random Effects** | | | | |
| σ^2^ | 0.30 | | | |
| τ_00_ _ID_ | 0.57 | | | |
| τ_11_ _ID.years_ | 0.02 | | | |
| ρ_01_ _ID_ | -0.12 | | | |
| ICC | 0.71 | | | |
| N _ID_ | 160 | | | |
| Observations | 685 | | | |
| Marginal R^2^ / Conditional R^2^ | 0.078 / 0.732 | | | |

Figure 2: Rate of change in story recall as a function of AB42/40 levels. Both the biomarker and the cognition scores were z-scored to baseline. Lines represent the mean value of the biomarker (blue line), 1 standard deviation below the mean (red line) and 1 standard deviation above the mean (green line).

|  | **Paired Associates** | | | |
| --- | --- | --- | --- | --- |
| *Predictors* | *Estimates* | *CI* | *p* | *df* |
| (Intercept) | 1.89 | -0.62 – 4.39 | 0.141 | 663.00 |
| Aβ42/ Aβ40 | 0.00 | -0.15 – 0.16 | 0.961 | 663.00 |
| years | -0.00 | -0.04 – 0.03 | 0.854 | 663.00 |
| bl age | -0.04 | -0.07 – -0.01 | **0.004** | 663.00 |
| EDUC | 0.04 | -0.02 – 0.10 | 0.171 | 663.00 |
| GENDER | 0.39 | 0.10 – 0.68 | **0.009** | 663.00 |
| Aβ42/ Aβ40 × years | 0.02 | -0.01 – 0.06 | 0.159 | 663.00 |
| **Random Effects** | | | | |
| σ^2^ | 0.35 | | | |
| τ_00_ _ID_ | 0.74 | | | |
| τ_11_ _ID.years_ | 0.01 | | | |
| ρ_01_ _ID_ | -0.11 | | | |
| ICC | 0.70 | | | |
| N _ID_ | 160 | | | |
| Observations | 674 | | | |
| Marginal R^2^ / Conditional R^2^ | 0.087 / 0.723 | | | |

Figure 3: Rate of change in paired associates recall as a function of AB42/40 levels. Both the biomarker and the cognition scores were z-scored to baseline. Lines represent the mean value of the biomarker (blue line), 1 standard deviation below the mean (red line) and 1 standard deviation above the mean (green line).

|  | **pacc fr** | | | |
| --- | --- | --- | --- | --- |
| *Predictors* | *Estimates* | *CI* | *p* | *df* |
| (Intercept) | 2.08 | 0.42 – 3.75 | **0.014** | 681.00 |
| Aβ42/ Aβ40 | 0.02 | -0.08 – 0.11 | 0.750 | 681.00 |
| years | -0.04 | -0.06 – -0.02 | **0.001** | 681.00 |
| bl age | -0.04 | -0.06 – -0.03 | **<0.001** | 681.00 |
| EDUC | 0.05 | 0.01 – 0.08 | **0.018** | 681.00 |
| GENDER | 0.28 | 0.08 – 0.47 | **0.005** | 681.00 |
| Aβ42/ Aβ40 × years | 0.03 | 0.01 – 0.05 | **0.007** | 681.00 |
| **Random Effects** | | | | |
| σ^2^ | 0.08 | | | |
| τ_00_ _ID_ | 0.33 | | | |
| τ_11_ _ID.years_ | 0.01 | | | |
| ρ_01_ _ID_ | 0.41 | | | |
| ICC | 0.87 | | | |
| N _ID_ | 161 | | | |
| Observations | 692 | | | |
| Marginal R^2^ / Conditional R^2^ | 0.153 / 0.887 | | | |

Figure 4: Rate of change in PACC with free recall as a function of AB42/40 levels. Both the biomarker and the cognition scores were z-scored to baseline. Lines represent the mean value of the biomarker (blue line), 1 standard deviation below the mean (red line) and 1 standard deviation above the mean (green line).

|  | **pacc fr pr** | | | |
| --- | --- | --- | --- | --- |
| *Predictors* | *Estimates* | *CI* | *p* | *df* |
| (Intercept) | 1.99 | 0.36 – 3.62 | **0.017** | 681.00 |
| Aβ42/ Aβ40 | 0.04 | -0.06 – 0.13 | 0.452 | 681.00 |
| years | -0.02 | -0.05 – -0.00 | **0.033** | 681.00 |
| bl age | -0.04 | -0.06 – -0.02 | **<0.001** | 681.00 |
| EDUC | 0.04 | 0.01 – 0.08 | **0.025** | 681.00 |
| GENDER | 0.29 | 0.10 – 0.48 | **0.003** | 681.00 |
| Aβ42/ Aβ40 × years | 0.02 | -0.00 – 0.04 | 0.105 | 681.00 |
| **Random Effects** | | | | |
| σ^2^ | 0.07 | | | |
| τ_00_ _ID_ | 0.32 | | | |
| τ_11_ _ID.years_ | 0.01 | | | |
| ρ_01_ _ID_ | 0.26 | | | |
| ICC | 0.88 | | | |
| N _ID_ | 161 | | | |
| Observations | 692 | | | |
| Marginal R^2^ / Conditional R^2^ | 0.153 / 0.897 | | | |

Figure 5: Rate of change in the PACC with free recall and story recall as a function of AB42/40 levels. Both the biomarker and the cognition scores were z-scored to baseline. Lines represent the mean value of the biomarker (blue line), 1 standard deviation below the mean (red line) and 1 standard deviation above the mean (green line).

|  | **Free Recall** | | | |
| --- | --- | --- | --- | --- |
| *Predictors* | *Estimates* | *CI* | *p* | *df* |
| (Intercept) | 1.01 | -1.36 – 3.38 | 0.403 | 668.00 |
| ptau217 | -0.16 | -0.31 – -0.01 | **0.034** | 668.00 |
| years | -0.01 | -0.05 – 0.02 | 0.504 | 668.00 |
| bl age | -0.02 | -0.04 – 0.01 | 0.267 | 668.00 |
| EDUC | -0.03 | -0.08 – 0.03 | 0.363 | 668.00 |
| GENDER | 0.40 | 0.12 – 0.67 | **0.005** | 668.00 |
| ptau217 × years | -0.08 | -0.13 – -0.04 | **<0.001** | 668.00 |
| **Random Effects** | | | | |
| σ^2^ | 0.26 | | | |
| τ_00_ _ID_ | 0.62 | | | |
| τ_11_ _ID.years_ | 0.02 | | | |
| ρ_01_ _ID_ | 0.07 | | | |
| ICC | 0.76 | | | |
| N _ID_ | 160 | | | |
| Observations | 679 | | | |
| Marginal R^2^ / Conditional R^2^ | 0.118 / 0.786 | | | |

Figure 6: Rate of change in FCSRT free recall as a function of ptau 217 levels Both the biomarker and the cognition scores were z-scored to baseline. Lines represent the mean value of the biomarker (blue line), 1 standard deviation below the mean (red line) and 1 standard deviation above the mean (green line).

|  | **Paragraph Recall** | | | |
| --- | --- | --- | --- | --- |
| *Predictors* | *Estimates* | *CI* | *p* | *df* |
| (Intercept) | 0.76 | -1.41 – 2.93 | 0.492 | 674.00 |
| ptau217 | -0.20 | -0.34 – -0.06 | **0.004** | 674.00 |
| years | 0.03 | -0.01 – 0.06 | 0.192 | 674.00 |
| bl age | -0.02 | -0.05 – 0.00 | 0.063 | 674.00 |
| EDUC | 0.03 | -0.02 – 0.08 | 0.222 | 674.00 |
| GENDER | 0.35 | 0.10 – 0.60 | **0.006** | 674.00 |
| ptau217 × years | -0.03 | -0.07 – 0.01 | 0.150 | 674.00 |
| **Random Effects** | | | | |
| σ^2^ | 0.30 | | | |
| τ_00_ _ID_ | 0.54 | | | |
| τ_11_ _ID.years_ | 0.02 | | | |
| ρ_01_ _ID_ | -0.19 | | | |
| ICC | 0.69 | | | |
| N _ID_ | 160 | | | |
| Observations | 685 | | | |
| Marginal R^2^ / Conditional R^2^ | 0.130 / 0.729 | | | |

Figure 7: Rate of change in story recall as a function of ptau 217 levels. Both the biomarker and the cognition scores were z-scored to baseline. Lines represent the mean value of the biomarker (blue line), 1 standard deviation below the mean (red line) and 1 standard deviation above the mean (green line).

|  | **Paired Associates** | | | |
| --- | --- | --- | --- | --- |
| *Predictors* | *Estimates* | *CI* | *p* | *df* |
| (Intercept) | 1.04 | -1.39 – 3.47 | 0.400 | 663.00 |
| ptau217 | -0.17 | -0.33 – -0.01 | **0.034** | 663.00 |
| years | -0.00 | -0.04 – 0.03 | 0.874 | 663.00 |
| bl age | -0.03 | -0.06 – -0.00 | **0.035** | 663.00 |
| EDUC | 0.04 | -0.02 – 0.09 | 0.202 | 663.00 |
| GENDER | 0.40 | 0.12 – 0.68 | **0.005** | 663.00 |
| ptau217 × years | -0.07 | -0.11 – -0.02 | **0.002** | 663.00 |
| **Random Effects** | | | | |
| σ^2^ | 0.35 | | | |
| τ_00_ _ID_ | 0.69 | | | |
| τ_11_ _ID.years_ | 0.01 | | | |
| ρ_01_ _ID_ | -0.18 | | | |
| ICC | 0.68 | | | |
| N _ID_ | 160 | | | |
| Observations | 674 | | | |
| Marginal R^2^ / Conditional R^2^ | 0.147 / 0.724 | | | |

Figure 8: Rate of change in paired associates recall as a function of ptau 217 levels. Both the biomarker and the cognition scores were z-scored to baseline. Lines represent the mean value of the biomarker (blue line), 1 standard deviation below the mean (red line) and 1 standard deviation above the mean (green line).

|  | **pacc fr** | | | |
| --- | --- | --- | --- | --- |
| *Predictors* | *Estimates* | *CI* | *p* | *df* |
| (Intercept) | 1.98 | 0.31 – 3.65 | **0.020** | 681.00 |
| ptau217 | -0.07 | -0.17 – 0.02 | 0.140 | 681.00 |
| years | -0.04 | -0.06 – -0.02 | **<0.001** | 681.00 |
| bl age | -0.04 | -0.06 – -0.02 | **<0.001** | 681.00 |
| EDUC | 0.05 | 0.01 – 0.08 | **0.019** | 681.00 |
| GENDER | 0.28 | 0.08 – 0.47 | **0.005** | 681.00 |
| ptau217 × years | -0.07 | -0.09 – -0.05 | **<0.001** | 681.00 |
| **Random Effects** | | | | |
| σ^2^ | 0.08 | | | |
| τ_00_ _ID_ | 0.33 | | | |
| τ_11_ _ID.years_ | 0.01 | | | |
| ρ_01_ _ID_ | 0.36 | | | |
| ICC | 0.86 | | | |
| N _ID_ | 161 | | | |
| Observations | 692 | | | |
| Marginal R^2^ / Conditional R^2^ | 0.222 / 0.889 | | | |

Figure 9: Rate of change in PACC with free recall as a function of ptau 217 levels. Both the biomarker and the cognition scores were z-scored to baseline. Lines represent the mean value of the biomarker (blue line), 1 standard deviation below the mean (red line) and 1 standard deviation above the mean (green line).

|  | **pacc fr pr** | | | |
| --- | --- | --- | --- | --- |
| *Predictors* | *Estimates* | *CI* | *p* | *df* |
| (Intercept) | 1.83 | 0.21 – 3.45 | **0.027** | 681.00 |
| ptau217 | -0.10 | -0.20 – -0.00 | **0.043** | 681.00 |
| years | -0.02 | -0.04 – -0.00 | **0.021** | 681.00 |
| bl age | -0.04 | -0.06 – -0.02 | **<0.001** | 681.00 |
| EDUC | 0.04 | 0.01 – 0.08 | **0.025** | 681.00 |
| GENDER | 0.29 | 0.11 – 0.48 | **0.002** | 681.00 |
| ptau217 × years | -0.06 | -0.08 – -0.04 | **<0.001** | 681.00 |
| **Random Effects** | | | | |
| σ^2^ | 0.07 | | | |
| τ_00_ _ID_ | 0.31 | | | |
| τ_11_ _ID.years_ | 0.01 | | | |
| ρ_01_ _ID_ | 0.18 | | | |
| ICC | 0.87 | | | |
| N _ID_ | 161 | | | |
| Observations | 692 | | | |
| Marginal R^2^ / Conditional R^2^ | 0.237 / 0.898 | | | |

Figure 10: Rate of change in PACC with free recall and story recall as a function of ptau 217 levels. Both the biomarker and the cognition scores were z-scored to baseline. Lines represent the mean value of the biomarker (blue line), 1 standard deviation below the mean (red line) and 1 standard deviation above the mean (green line).

|  | **Free Recall** | | | |
| --- | --- | --- | --- | --- |
| *Predictors* | *Estimates* | *CI* | *p* | *df* |
| (Intercept) | 2.03 | -0.71 – 4.77 | 0.147 | 668.00 |
| NfL | 0.06 | -0.10 – 0.23 | 0.460 | 668.00 |
| years | -0.01 | -0.04 – 0.03 | 0.748 | 668.00 |
| bl age | -0.03 | -0.06 – 0.00 | 0.069 | 668.00 |
| EDUC | -0.02 | -0.08 – 0.03 | 0.408 | 668.00 |
| GENDER | 0.39 | 0.11 – 0.68 | **0.007** | 668.00 |
| NfL × years | -0.03 | -0.07 – 0.00 | 0.063 | 668.00 |
| **Random Effects** | | | | |
| σ^2^ | 0.27 | | | |
| τ_00_ _ID_ | 0.65 | | | |
| τ_11_ _ID.years_ | 0.02 | | | |
| ρ_01_ _ID_ | 0.16 | | | |
| ICC | 0.77 | | | |
| N _ID_ | 160 | | | |
| Observations | 679 | | | |
| Marginal R^2^ / Conditional R^2^ | 0.060 / 0.787 | | | |

Figure 11: Rate of change in FCSRT free recall as a function of NfL levels. Both the biomarker and the cognition scores were z-scored to baseline. Lines represent the mean value of the biomarker (blue line), 1 standard deviation below the mean (red line) and 1 standard deviation above the mean (green line).

|  | **Paragraph Recall** | | | |
| --- | --- | --- | --- | --- |
| *Predictors* | *Estimates* | *CI* | *p* | *df* |
| (Intercept) | 1.98 | -0.54 – 4.51 | 0.124 | 674.00 |
| NfL | 0.07 | -0.09 – 0.23 | 0.373 | 674.00 |
| years | 0.03 | -0.01 – 0.06 | 0.178 | 674.00 |
| bl age | -0.04 | -0.07 – -0.01 | **0.006** | 674.00 |
| EDUC | 0.04 | -0.02 – 0.09 | 0.180 | 674.00 |
| GENDER | 0.34 | 0.08 – 0.60 | **0.010** | 674.00 |
| NfL × years | -0.02 | -0.06 – 0.02 | 0.352 | 674.00 |
| **Random Effects** | | | | |
| σ^2^ | 0.30 | | | |
| τ_00_ _ID_ | 0.59 | | | |
| τ_11_ _ID.years_ | 0.02 | | | |
| ρ_01_ _ID_ | -0.14 | | | |
| ICC | 0.71 | | | |
| N _ID_ | 160 | | | |
| Observations | 685 | | | |
| Marginal R^2^ / Conditional R^2^ | 0.077 / 0.732 | | | |

Figure 12: Rate of change in story recall as a function of NfL levels. Both the biomarker and the cognition scores were z-scored to baseline. Lines represent the mean value of the biomarker (blue line), 1 standard deviation below the mean (red line) and 1 standard deviation above the mean (green line).

|  | **Paired Associates** | | | |
| --- | --- | --- | --- | --- |
| *Predictors* | *Estimates* | *CI* | *p* | *df* |
| (Intercept) | 2.79 | -0.02 – 5.59 | 0.052 | 663.00 |
| NfL | 0.15 | -0.02 – 0.32 | 0.089 | 663.00 |
| years | 0.00 | -0.03 – 0.04 | 0.981 | 663.00 |
| bl age | -0.06 | -0.09 – -0.02 | **0.001** | 663.00 |
| EDUC | 0.04 | -0.02 – 0.10 | 0.180 | 663.00 |
| GENDER | 0.38 | 0.09 – 0.67 | **0.010** | 663.00 |
| NfL × years | -0.03 | -0.07 – 0.00 | 0.053 | 663.00 |
| **Random Effects** | | | | |
| σ^2^ | 0.35 | | | |
| τ_00_ _ID_ | 0.73 | | | |
| τ_11_ _ID.years_ | 0.01 | | | |
| ρ_01_ _ID_ | -0.11 | | | |
| ICC | 0.70 | | | |
| N _ID_ | 160 | | | |
| Observations | 674 | | | |
| Marginal R^2^ / Conditional R^2^ | 0.091 / 0.723 | | | |

Figure 13: Rate of change in paired associates recall as a function of NfL levels. Both the biomarker and the cognition scores were z-scored to baseline. Lines represent the mean value of the biomarker (blue line), 1 standard deviation below the mean (red line) and 1 standard deviation above the mean (green line).

|  | **pacc fr** | | | |
| --- | --- | --- | --- | --- |
| *Predictors* | *Estimates* | *CI* | *p* | *df* |
| (Intercept) | 1.96 | 0.08 – 3.85 | **0.041** | 681.00 |
| NfL | -0.03 | -0.14 – 0.09 | 0.648 | 681.00 |
| years | -0.04 | -0.06 – -0.01 | **0.002** | 681.00 |
| bl age | -0.04 | -0.07 – -0.02 | **<0.001** | 681.00 |
| EDUC | 0.05 | 0.01 – 0.09 | **0.017** | 681.00 |
| GENDER | 0.28 | 0.08 – 0.47 | **0.006** | 681.00 |
| NfL × years | -0.02 | -0.04 – -0.00 | **0.049** | 681.00 |
| **Random Effects** | | | | |
| σ^2^ | 0.08 | | | |
| τ_00_ _ID_ | 0.33 | | | |
| τ_11_ _ID.years_ | 0.01 | | | |
| ρ_01_ _ID_ | 0.39 | | | |
| ICC | 0.87 | | | |
| N _ID_ | 161 | | | |
| Observations | 692 | | | |
| Marginal R^2^ / Conditional R^2^ | 0.152 / 0.888 | | | |

Figure 14: Rate of change in PACC with free recall as a function of NfL levels. Both the biomarker and the cognition scores were z-scored to baseline. Lines represent the mean value of the biomarker (blue line), 1 standard deviation below the mean (red line) and 1 standard deviation above the mean (green line).

|  | **pacc fr pr** | | | |
| --- | --- | --- | --- | --- |
| *Predictors* | *Estimates* | *CI* | *p* | *df* |
| (Intercept) | 2.05 | 0.20 – 3.89 | **0.030** | 681.00 |
| NfL | -0.00 | -0.11 – 0.11 | 0.951 | 681.00 |
| years | -0.02 | -0.04 – -0.00 | **0.048** | 681.00 |
| bl age | -0.04 | -0.07 – -0.02 | **<0.001** | 681.00 |
| EDUC | 0.04 | 0.01 – 0.08 | **0.023** | 681.00 |
| GENDER | 0.30 | 0.10 – 0.49 | **0.002** | 681.00 |
| NfL × years | -0.02 | -0.04 – -0.00 | **0.036** | 681.00 |
| **Random Effects** | | | | |
| σ^2^ | 0.07 | | | |
| τ_00_ _ID_ | 0.32 | | | |
| τ_11_ _ID.years_ | 0.01 | | | |
| ρ_01_ _ID_ | 0.26 | | | |
| ICC | 0.88 | | | |
| N _ID_ | 161 | | | |
| Observations | 692 | | | |
| Marginal R^2^ / Conditional R^2^ | 0.157 / 0.899 | | | |

Figure 15: Rate of change in PACC with free recall and story recall as a function of NfL levels. Both the biomarker and the cognition scores were z-scored to baseline. Lines represent the mean value of the biomarker (blue line), 1 standard deviation below the mean (red line) and 1 standard deviation above the mean (green line).
